# Unraveling the dynamics of loneliness and cognition in late life: a cross-lagged panel model

**DOI:** 10.3389/fpubh.2024.1425403

**Published:** 2024-08-07

**Authors:** Elnaz Abaei, Peter Martin

**Affiliations:** Human Development and Family Studies, Iowa State University, Ames, IA, United States

**Keywords:** cognition, loneliness, centenarian, Health and Retirement Study (HRS), demographic factors

## Abstract

**Introduction:**

Loneliness and cognitive decline are pressing concerns among older adults, yet little research has explored cognition as a predictor of loneliness. This study investigates the dynamic relationship between loneliness and cognitive function in older adults using the random intercept cross-lagged panel model (RI-CLPM).

**Methods:**

Data were drawn from Waves 9–14 of the Health and Retirement Study (HRS), encompassing 8,473 individuals aged 65 years and older. Loneliness was assessed using the UCLA Loneliness Scale, and cognitive function was measured using immediate and delayed word recall and serial 7s from the HRS RAND file. Age, gender, education, marital status, self-health report, and depression were included as covariates. Using M*plus*, we computed RI-CLPMs. The first three models were conducted on loneliness and cognitive functions. Then unconditional RI-CLPMs with no exogenous predictors were computed.

**Results:**

Three conditional model results showed that age, gender, marital status, self-health report, and depression were significantly associated with loneliness in the first wave, but only age and self-health report were significantly associated with immediate and delayed word recall at the first wave, not with serial 7s. For carry-over effects, loneliness showed significant positive associations across consecutive waves, but cognitive functions showed significant positive associations just in the last two waves. Some spill-over effects were found between loneliness and cognitive functions. For within-person effects, although initially non-significant, a negative association between loneliness and immediate and delayed word recall emerged in later waves (11–12 and 13–14). The conditional models indicated that older age, not being married, male gender, low self-reported health, and high depression levels were positively associated with loneliness. However, only older age and lower self-reported health were positively linked to cognitive functions.

**Discussion:**

This study underscores the link between loneliness and cognitive function decline in older adults, emphasizing the need to address loneliness to potentially reduce cognitive decline. Insights into demographic predictors of loneliness and cognitive function could inform targeted interventions for promoting successful aging.

## Introduction

Loneliness is a critical issue for older adults, who have lost many of their contemporaries. Several studies have explored various factors that can influence the loneliness experienced by older adults. Perlman and Peplau (1) defined loneliness as “the unpleasant experience that occurs when a person’s network of social relationships is deficient in some important way, either quantitatively or qualitatively” (p. 31). This definition treats loneliness as a unidimensional concept, varying primarily in its intensity of experience. Kim et al. (2) suggested that loneliness might indirectly affect cognitive ability by indicating declining physical and psychiatric health directly related to cognitive function. These health factors could be key intervention targets for maintaining cognition among lonely older adults. Additionally, cognitive impairment can hinder maintaining friendships, communicating with others, and participating in social and leisure activities (3), making diminished cognition both a consequence and a potential indicator of loneliness.

The association between loneliness and cognitive performance might also flow in the opposite direction. Only a few studies have confirmed cognition as a predictor of loneliness [e.g., (4)]. In their research, Sutin et al. (5) found a robust association between loneliness and risk of all-cause and cause-specific dementia: Feeling lonely was associated with about 60% increased risk of incident all-cause dementia over up to nearly 16 years of follow-up. Martin et al. (6) compared centenarians from Swedish and Georgian samples, finding an association between lower cognitive functioning and increased loneliness only in the Swedish sample. Ayalon et al. (7) aimed to establish the relationship between loneliness and memory functioning using data from the Health and Retirement Study (2004, 2008, and 2012). Among 1,225 participants aged 50 and older, lower memory functioning preceded higher loneliness levels over 4 years. In another study, Wang et al. (8) found no significant association between loneliness and cognitive function decline in individuals aged 75 and over. This was after adjusting for cohort effects, follow-up time, sex, education, and interaction terms for sex, education, and time, indicating that loneliness did not significantly impact cognitive function in this demographic group. O’Luanaigh et al. (9) reported that self-reported loneliness was linked to deficits in psychomotor processing speed and delayed visual memory among individuals for a group of older adults with an average age of 76. In their study on 509 community-residing older adults (with a mean age of 72), Hayslip et al. (10) found that higher levels of emotional loneliness were associated with higher scores for general fluid (Gf) ability. Cachón-Alonso et al. (11) observed that higher loneliness predicted lower cognitive function over a 7-year follow-up among individuals aged 50 and older. In their study involving 14,199 Chinese individuals aged 68–105 years, Zhong et al. (12) noted that severe loneliness predicted poorer cognitive function at subsequent assessments, partially mediated by chronic conditions. Montoliu et al. (13) found no direct association between loneliness and cognitive performance in a sample of 86 older individuals. While there is research on the association between loneliness and cognition in older adults, there appears to be a shortage of specific studies focusing on the oldest-old population (aged 85 years and above) focusing on loneliness and cognition. Margrett et al. (14) examined 55 octogenarians and 77 centenarians, finding limited associations between executive control, cognitive functioning, and mental health indicators.

Various studies have demonstrated the impact of demographic factors on loneliness. For instance, Dahlberg et al. (15) conducted a study to explore the association between loneliness and demographic factors. The results did not reveal a significant association between age and loneliness. However, the study found lower education levels and widowhood were associated with increased feelings of loneliness. Although several studies have not found a strong correlation between age and loneliness, Heylen’s (16) study reported that higher age was significantly correlated with a lower risk of loneliness. The bivariate results from this study also revealed that women were less likely to experience loneliness than men, but gender did not affect the path model significantly. Another study (17) showed that loneliness would have a U-shaped relationship with age across middle and late adulthood. Bishop and Martin (18) indicated that higher educational attainment reduced loneliness by lowering neuroticism and stress. However, Chow et al. (19) found no significant correlation between age, gender, education, and loneliness.

Cognitive abilities can be categorized into distinct domains: attention, memory, executive function, language, and visuospatial abilities. Each domain exhibits quantifiable declines with advancing age (20). The Atherosclerosis Risk in Communities Study (21) showed that education level positively correlated with cognitive test performance. Older adults performed worse on recall tasks than on recognition tasks, according to an experiment (22) that compared young (mean age = 21 years) and older adults (mean age = 73 years) on cued-recall and recognition tests while carrying out a choice reaction-time task. The analysis also showed that recall requires more processing resources than recognition, and this effect increases with age.

This study investigated the relationships between loneliness, cognitive function, and demographic variables across wave 9 (2008) to wave 14 (2018) for older adults from the Health and Retirement Study (HRS). When examining longitudinal data, it is necessary to recognize that occurrences are nested within individuals. This understanding emphasizes the necessity of distinguishing between within-person changes and the between-person differences. Computing a RI-CLMP allows for such a distinction by integrating a random intercept (23). We formulated two main research questions for our study: The first one is to understand how demographic factors such as age, gender, education, marital status, self-health report, and depression impact the loneliness of participants. To test this, we hypothesized that participants who were older, unmarried females and had lower education levels with low self-health report and high levels of depression were more likely to experience loneliness. The second research question was to examine the relationship between cognition and loneliness over time. Our hypothesis was that participants with lower levels of cognitive function were more likely to feel lonely.

## Materials and methods

### Participants

Data for this study come from the Health and Retirement Study (HRS). Created in 1990, the HRS is a national longitudinal panel study of the economic, health, marital, and family status of approximately 20,000 people over 50 years of age and their spouses. In this study, we included loneliness and cognition variables from the six waves of the study, which are waves 9 (2008), wave 10 (2010), 11 (2012), wave 12 (2014), 13 (2016), and 14 (2018). Furthermore, we investigated the effects of age, education, and gender. Because loneliness was only assessed for half of the sample for each wave, we pooled waves 9–10, 11–12, and 13–14 to increase the sample size. In this study, we only included individuals with 65 age and older, so our analytic sample is *N* = 8,473 (age mean = 74.90).

### Measures

#### Loneliness

HRS measured overall loneliness using the UCLA Loneliness Scale (24). Respondents were asked to 11 items, and rated their experiences on a three-point scale ranging from 1 = *often* to 3 = *hardly ever or never*. After four negatively worded items were reverse-coded, an overall loneliness score was calculated by averaging the scores of the 11 items.

#### Cognitive function

The cognitive performance tests that were conducted in the HRS consisted of various tasks such as immediate and delayed free recall, serial 7s, counting backwards from 20, naming the US president and vice president by their last names, naming two objects (scissors and cactus), and providing the date, including the month, day, year, and day of the week (25). In this study, we focused on immediate and delayed word recall (IWR and DWR) and serial 7s to assess the participants’ cognitive abilities. In the Health and Retirement Study (HRS), immediate and delayed word recall tests measure different aspects of memory function. Immediate word recall assesses short-term memory by asking participants to recall a list of words immediately after hearing them, reflecting their capacity for immediate memory encoding and retrieval. On the other hand, delayed word recall assesses long-term memory by asking participants to recall the same list of words after a delay, indicating their ability to retain and retrieve information over time. The serial 7s test is also included to measure attention and working memory; participants are asked to subtract seven from 100 and 7 from each subsequent result. The questions asked between the immediate and delayed recall tasks varied somewhat across different survey waves. For instance, in 1998, the CESD depression items, backward count, and serial 7’s were administered between the two recall tasks. In contrast 1996, only cognition-related items such as date naming, backward count, object naming, and President/Vice President naming were administered between the two recall tasks (26).

### Design and analyses

Descriptive analyses were employed to calculate means and standard deviations for the variables. Bivariate correlations were then computed among loneliness, cognition function, and demographic factors (age, gender, education, marital status, self-health report, and depression).

We used the RI-CLPM modeling strategy (27) proposed to investigate the association between loneliness and cognitive function. First, we begin by considering the relationship between loneliness and cognitive functions. We fit three separate RI-CLPMs to examine the relationships between:

1. Loneliness and immediate word recall,
2. Loneliness and delayed word recall,
3. Loneliness and serial 7s.

**Figure 1:**
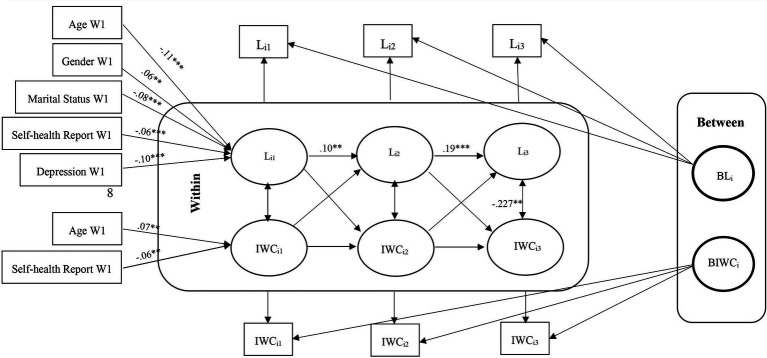
RI-CLPM Loneliness-Immediate word recall (IWC) Model. L_i1_, Loneliness at waves 9 and 10; L_i2_, Loneliness at waves 11 and 12; L_i3_, Loneliness at waves 13 and 14; IWC_i1_, Immediate word recall at waves 9 and 10; IWC_i2_, Immediate word recall at waves 11 and 12; IWC_i3_, Immediate word recall at waves 13 and 14; ***Significant at *p* < 0.001, **Significant at *p* < 0.01, *Significant at *p* < 0.05.

**Figure 2:**
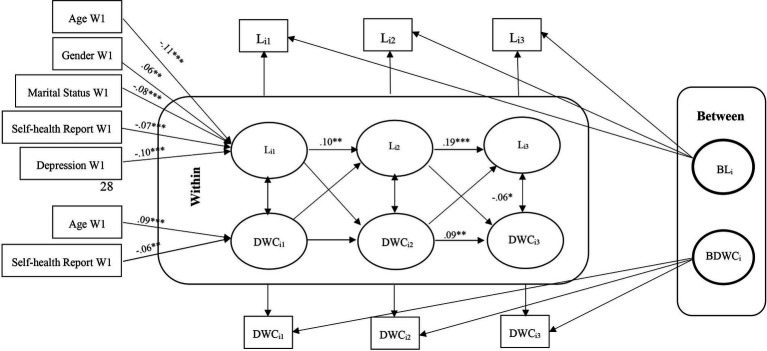
RI-CLPM Loneliness- Delayed word recall (DWC) Model. L_i1_, Loneliness at waves 9 and 10; L_i2_, Loneliness at waves 11 and 12; L_i3_, Loneliness at waves 13 and 14; DWC_i1_, Delayed word recall at waves 9 and 10; DWC_i2_, Delayed word recall at waves 11 and 12; DWC_i3_, Delayed word recall at waves 13 and 14; ***Significant at *p* < 0.001, **Significant at *p* < 0.01, *Significant at *p* < 0.05.

**Figure 3:**
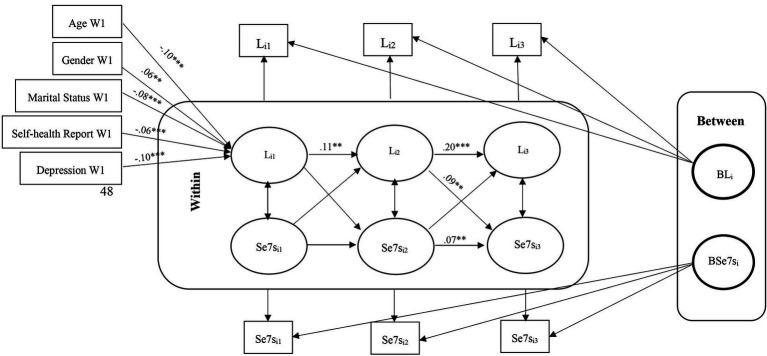
RI-CLPM Loneliness- Serial 7s Model. L_i1_, Loneliness at waves 9 and 10; L_i2_, Loneliness at waves 11 and 12; L_i3_, Loneliness at waves 13 and 14; Se7s_i1_, Serial 7s at waves 9 and 10; Se7s_i2_, Serial 7s at waves 11 and 12; Se7s_i3_, Serial 7s at waves 13 and 14; ***Significant at *p* < 0.001, **Significant at *p* < 0.01, *Significant at *p* < 0.05.

**Figure 4:**
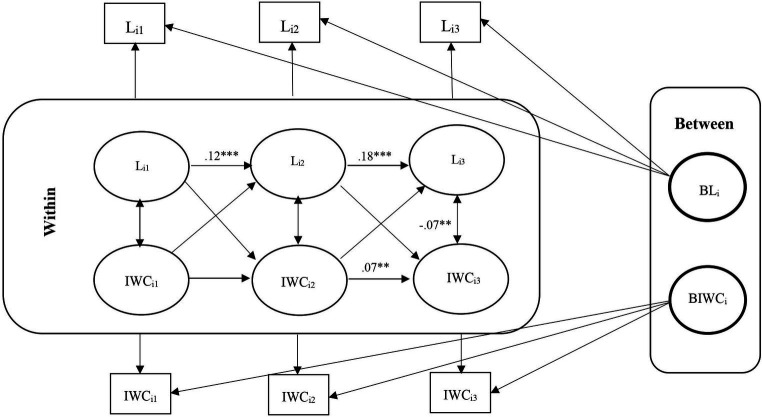
Unconditional RI-CLPM Loneliness-Immediate word recall (IWC) Model. L_i1_, Loneliness at waves 9 and 10; L_i2_, Loneliness at waves 11 and 12; L_i3_, Loneliness at waves 13 and 14; IWC_i1_, Immediate word recall at waves 9 and 10; IWC_i2_, Immediate word recall at waves 11 and 12; IWC_i3_, Immediate word recall at waves 13 and 14; ***Significant at *p* < 0.001, **Significant at *p* < 0.01, *Significant at *p* < 0.05.

**Figure 5:**
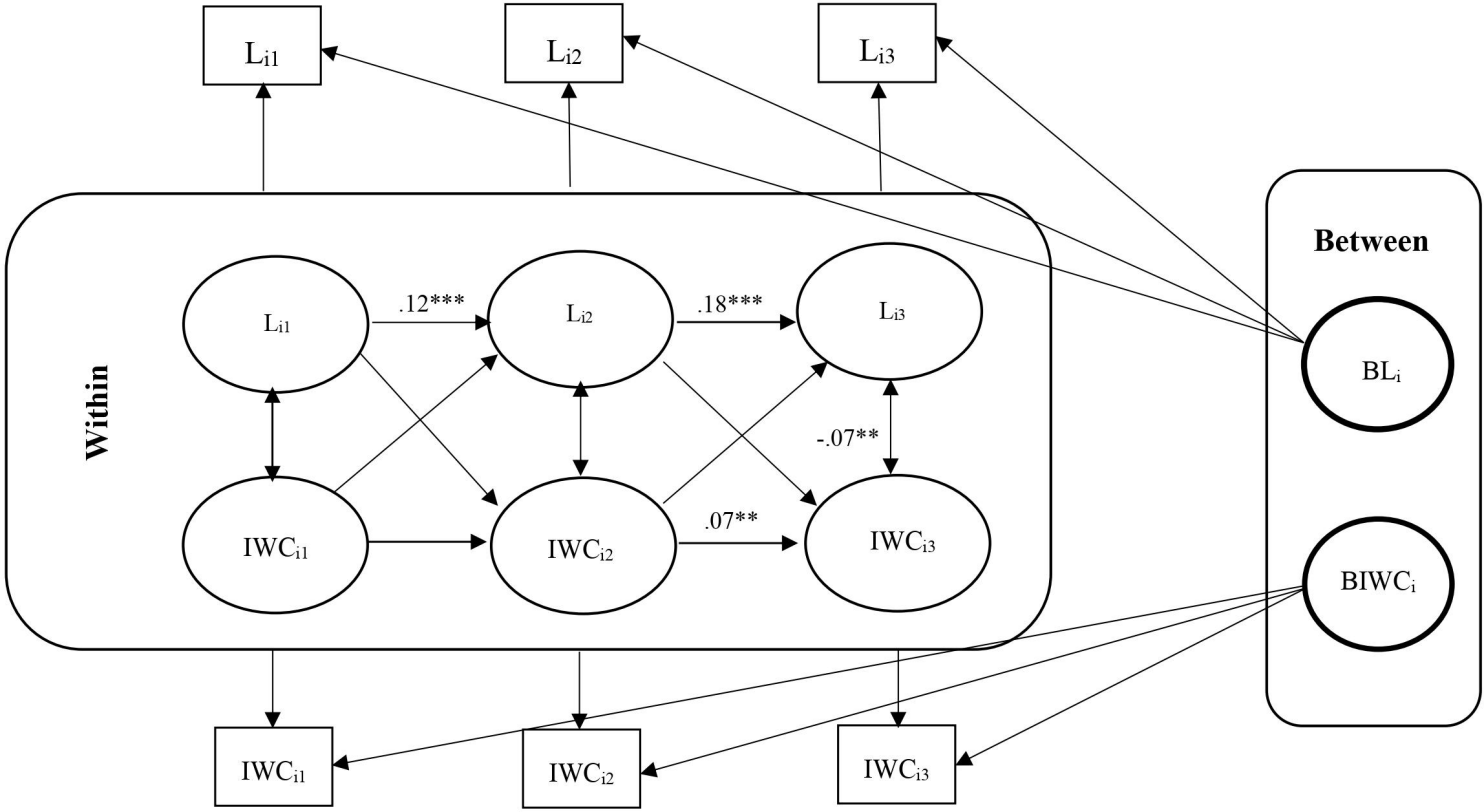
Unconditional RI-CLPM Loneliness- Delayed word recall (DWC) Model. L_i2_, Loneliness at waves 9 and 10; L_i2_, Loneliness at waves 11 and 12; L_i3_, Loneliness at waves 13 and 14; DWC_i1_, Delayed word recall at waves 9 and 10; DWC_i2_, Delayed word recall at waves 11 and 12; DWC_i3_, Delayed word recall at waves 13 and 14; ***Significant at *p* < 0.001, **Significant at *p* < 0.01, *Significant at *p* < 0.05.

**Figure 6:**
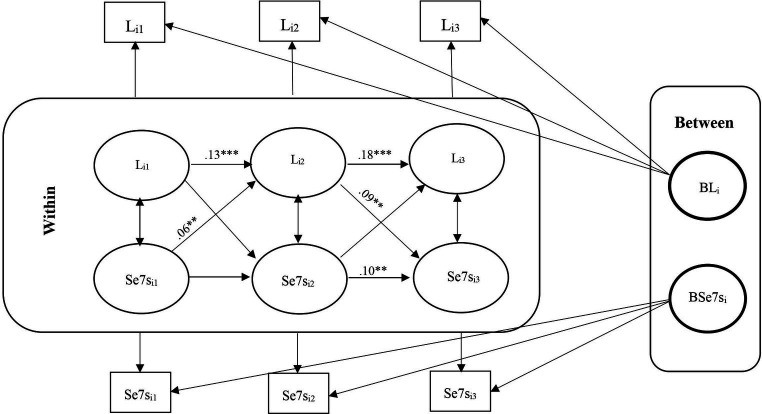
Unconditional RI-CLPM Standardized Loneliness-Serial 7s Model Results. L_i1_, Loneliness at waves 9 and 10; L_i2_, Loneliness at waves 11 and 12; L_i2_, Loneliness at waves 13 and 14; Se7s_i1_, Serial 7s at waves 9 and 10; Se7_i2_, Serial 7s at waves 11 and 12; Se7_i3_, Serial 7s at waves 13 and 14; ***Significant at *p* < 0.001, **Significant at *p* < 0.01, *Significant at *p* < 0.05.

These models help us understand whether deviations from expected loneliness scores predict deviations from expected cognitive function scores in subsequent waves and vice versa. Second, we modeled unconditional models without covariates by considering the relationship between loneliness and cognitive function. We fit three model in which the means of each variable were constrained over time, while the covariance structure was unconstrained. Models in which the group means do not change over time facilitate interpretation, although time-invariant means are not a prerequisite for the models considered here. The fit of these models were assessed to determine if any adjustments were necessary. Figures 1–6 represent the basic structure and components of the RI-CLPMs.

We restricted our dataset to include participants aged 65 and older, aligning with the demographic focus of our investigation. Additionally, we filtered the data to only include cases with available data on the loneliness variable.

Model fit was assessed using various indices, including χ^2^, Comparative Fit Index (CFI), Tucker-Lewis Index (TLI), and Root Mean Square Error of Approximation (RMSEA). Acceptable model fit is indicated by CFI values exceeding 0.95 and RMSEA values at or below 0.05, suggesting strong alignment between the proposed model and the observed data (28).

### Statistical power analysis

To ensure the reliability of our results, we conducted a power analysis for our structural equation model. This analysis involved 13 degrees of freedom and a sample size of 8,473 participants. Following the guidelines from McCallum et al. (29), we defined the parameters for Root Mean Square Error of Approximation (RMSEA) as H0 = 0.05 and H1 = 0.09. The analysis indicated a power of 1.00 for testing close fit, which suggests a high likelihood of detecting genuine relationships and confirms that our sample size is adequate for the specified model.

## Results

### Descriptive analysis

From 17,217 individuals (age mean = 69.20), we only included the participants who were 65 years of age and older at waves 9–10 (i.e., in 2008 and 2010). So, the total analytic sample of this study is *N* = 8,473 (age mean = 74.90). At baseline, the respondents identified themselves as men (43.0%) or women (57.0%). Among the respondents, 85.7% self-identified as white, whereas the remaining individuals identified as Black/African American or belonging to other ethnicities. On average, the participants’ education level was slightly above high school graduation, and 58.3% of respondents reported being married (Table 1).

**Table 1:** Demographic characteristics.

Variable	*n*	*M*	SD	%
Age (Waves 9–10)		74.90	7.013	
Gender				
Male	3,644			43.0
Female	4,829			57.0
Ethnicity				
White	7,264			85.7
Black/African American	945			11.2
Other	264			3.1
Education				
Lt High-school	1,741			20.6
GED	386			4.6
High-school graduate	2,859			33.7
Some college	1,790			21.1
College and above	1,696			20.0
Marital status				
Married	4,945			58.3
Not married	3,538			41.7

### Measures score ranges

We used the UCLA Loneliness Scale (24) to measure participants loneliness. The loneliness scores ranged from 11 to 33. In Wave 9–10, 8,483 participants had an average score of 16.46 (*SD* = 4.563). In Wave 11–12, 7,835 participants recorded a similar average loneliness score of 16.55, with slightly higher variability (*SD* = 4.574). Wave 13–14, with 6,198 participants, showed a somewhat higher average loneliness score of 16.67, with comparable variability (*SD* = 4.676). Coefficient alpha values ranged from 0.88 to 0.89.

We also focused on immediate and delayed word recall and serial 7s variables to assess the participants’ cognitive abilities. Across three waves, the immediate and delayed word recall and serial 7s scores ranged from 0 to 10 and 0 to 5, respectively. In Wave 9–10, with 6,889 participants, scores on the immediate and delayed word recall tasks were on average 5.22 (*SD* = 1.570) and 4.12 (*SD* = 1.863), respectively, and the serial 7s scores were on average 3.48 (*SD* = 1.653). In Wave 11–12, with 5,357 participants, a slightly lower mean score of 5.01 (*SD* = 1.602), 3.94 (*SD* = 1.91) and 3.48 (*SD* = 1.64) were recorded for immediate and delayed word recall and serial 7s, respectively. Wave 13–14, with 3,166 participants, showed a slightly lower mean score of 4.99 (*SD* = 1.58) for the immediate word recall variable and slightly higher mean score of 3.96 (*SD* = 1.88) and 3.51 (*SD* = 1.63) for the delayed word recall and serial 7s, respectively.

### Covariates in loneliness and cognitive function: correlations and associations

We included the covariates age, gender, education, marital status, self-health report, and depression in this research. Bivariate correlations revealed that older age was negatively correlated with cognitive functions, while loneliness was positively correlated with older age. Male gender and higher education were positively correlated with cognitive function and negatively correlated with loneliness. Marital status showed a negative correlation with loneliness, indicating that married individuals feel less lonely (Table 2). Additionally, self-health report was positively associated with loneliness and negatively with cognitive functions. Depressive symptoms was positively associated with loneliness and negatively with cognitive functions. All results showed that age, gender, marital status, self-health report, and depression were significantly associated with loneliness in the first wave, but only age and self-health report were significantly associated with immediate and delayed word recall at the first wave, but not with serial 7s. These findings highlight the complex interplay of demographic and health-related factors in understanding the relationships between loneliness and cognitive function over time (Table 3).

**Table 2:** Correlations between demographics, loneliness and cognitive variables.

Correlations																		
	1	2	3	4	5	6	7	8	9	10	11	12	13	14	15	16	17	18
1. Age (Wave 9–10)	1																	
2. Gender	0.019	1																
3. Education	−0.055^**^	−0.050^**^	1															
4. Marital status	−0.217^**^	−0.292^**^	0.112^**^	1														
5. Depression	0.046^**^	0.105^**^	−0.195^**^	−0.172^**^	1													
6. Self-health report	0.085^**^	−0.011	−0.252^**^	−0.092^**^	0.378^**^	1												
7. Loneliness W 9–10	0.059^**^	−0.038^**^	−0.153^**^	−0.141^**^	0.373^**^	0.249^**^	1											
8. Loneliness W 11–12	0.070^**^	−0.029^*^	−0.120^**^	−0.103^**^	0.317^**^	0.247^**^	0.612^**^	1										
9. Loneliness W 13–14	0.032	−0.006	−0.119^**^	−0.071^**^	0.263^**^	0.229^**^	0.566^**^	0.623^**^	1									
10. IWC W 9–10	−0.300^**^	0.178^**^	0.303^**^	0.054^**^	−0.167^**^	−0.194^**^	−0.150^**^	−0.132^**^	−0.114^**^	1								
11. IWC W 11–12	−0.301^**^	0.157^**^	0.293^**^	0.054^**^	−0.132^**^	−0.184^**^	−0.109^**^	−0.134^**^	−0.102^**^	0.511^**^	1							
12. IWC W 13–14	−0.242^**^	0.127^**^	0.283^**^	0.061^**^	−0.102^**^	−0.209^**^	−0.102^**^	−0.111^**^	−0.143^**^	0.477^**^	0.501^**^	1						
13. DWC W 9–10	−0.299^**^	0.152^**^	0.278^**^	0.045^**^	−0.154^**^	−0.188^**^	−0.145^**^	−0.121^**^	−0.097^**^	0.733^**^	0.461^**^	0.439^**^	1					
14. DWC W 11–12	−0.312^**^	0.141^**^	0.266^**^	0.055^**^	−0.128^**^	−0.191^**^	−0.107^**^	−0.126^**^	−0.097^**^	0.482^**^	0.747^**^	0.483^**^	0.532^**^	1				
15. DWC W 13–14	−0.261^**^	0.109^**^	0.271^**^	0.070^**^	−0.112^**^	−0.190^**^	−0.091^**^	−0.120^**^	−0.135^**^	0.439^**^	0.474^**^	0.732^**^	0.489^**^	0.537^**^	1			
16. Se7s W 9–10	−0.062^**^	−0.134^**^	0.383^**^	0.114^**^	−0.209^**^	−0.204^**^	−0.132^**^	−0.084^**^	−0.115^**^	0.309^**^	0.243^**^	0.240^**^	0.302^**^	0.254^**^	0.222^**^	1		
17. Se7s W 11–12	−0.073^**^	−0.133^**^	0.365^**^	0.117^**^	−0.190^**^	−0.204^**^	−0.104^**^	−0.095^**^	−0.104^**^	0.284^**^	0.311^**^	0.243^**^	0.267^**^	0.306^**^	0.227^**^	0.624^**^	1	
18. Se7s W 13–114	−0.028	−0.151^**^	0.366^**^	0.120^**^	−0.166^**^	−0.181^**^	−0.077^**^	−0.048^**^	−0.070^**^	0.243^**^	0.262^**^	0.295^**^	0.231^**^	0.254^**^	0.272^**^	0.607^**^	0.632^**^	1

**Correlation is significant at the 0.01 level (two-tailed).

*Correlation is significant at the 0.05 level (two-tailed).

Loneliness W9-10, Loneliness at waves 9 and 10; Loneliness W11-12, Loneliness at waves 11 and 12; Loneliness W13-14, Loneliness at waves 13 and 14; IWC W 9–10, Immediate word recall at waves 9 and 10; IWC W 11–12, Immediate word recall at waves 11 and 12; IWC W 13–14, Immediate word recall at waves 13 and 14; DWC W 9–10, Delayed word recall at waves 9 and 10; DWC W 11–12, Delayed word recall at waves 11 and 12; DWC W 13–14, Delayed word recall at waves 13 and 14; Se7s W 9–10, Serial 7s at waves 9 and 10; Se7s W 11–12, Serial 7s at waves 11 and 12; Se7s W 13–14, Serial 7s at waves 13 and 14.

**Table 3:** RI-CLPM Loneliness-Immediate Word Recall (IWC), Delayed Word Recall (DEC), and Serial 7s Models Results.

Standardized and unstandardized model results	*B*	SE	*β*
RI-CLPM Loneliness-Immediate word recall (IWC) Model			
Loneliness W 9–10 on Age	−0.11***	0.02	−5.13
Loneliness W 9–10 on Gender	−0.06**	0.02	−2.97
Loneliness W 9–10 on Education	−0.02	0.02	−1.26
Loneliness W 9–10 on Marital status	−0.08***	0.02	−3.97
Loneliness W 9–10 on Self-health report	−0.06**	0.02	−2.88
Loneliness W 9–10 on Depression	−0.10***	0.02	4.13
Loneliness W 11–12 on Loneliness W 9–10	0.10**	0.04	2.79
Loneliness W 11–12 on IWC W 9–10	−0.01	0.03	−0.43
Loneliness W 13–14 on Loneliness W 11–12	0.19***	0.03	6.17
Loneliness W 13–14 on IWC W 11–12	0.02	0.03	0.60
IWC W 9–10 on Age	0.07**	0.02	3.11
IWC W 9–10 on Gender	0.02	0.02	1.25
IWC W 9–10 on Education	−0.00	0.02	−0.21
IWC W 9–10 on Marital status	−0.01	0.02	−0.57
IWC W 9–10 on Self-health report	0.06**	0.02	2.79
IWC W 9–10 on Depression	−0.03	0.02	−1.54
IWC W 11–12 on IWC 9–10	0.02	0.03	0.47
IWC W 11–12 on Loneliness W 9–10	−0.02	0.04	−0.41
IWC W 13–14 on IWC W 11–12	0.05	0.03	1.53
IWC W 13–14 on Loneliness W 11–12	−0.01	0.03	−0.39
RI-CLPM Loneliness-Delayed word recall (DWC) Model			
Loneliness W 9–10 on Age	−0.11***	0.02	−5.07
Loneliness W 9–10 on Gender	−0.06**	0.02	−2.95
Loneliness W 9–10 on Education	−0.03	0.02	−1.29
Loneliness W 9–10 on Marital status	−0.08***	0.02	−3.96
Loneliness W 9–10 on Self-health report	−0.07**	0.02	−2.92
Loneliness W 9–10 on Depression	0.10***	0.02	4.18
Loneliness W 11–12 on Loneliness W 9–10	0.10**	0.04	2.76
Loneliness W 11–12 on DWC W 9–10	−0.01	0.03	−0.28
Loneliness W 13–14 on Loneliness W 11–12	0.19***	0.03	6.15
Loneliness W 13–14 on DWC W 11–12	0.02	0.03	0.74
DWC W 9–10 on Age	0.09***	0.02	4.12
DWC W 9–10 on Gender	0.01	0.02	0.42
DWC W 9–10 on Education	−0.00	0.02	−0.20
DWC W 9–10 on Marital status	−0.03	0.02	−1.64
DWC W 9–10 on Self-health report	0.06**	0.02	2.59
DWC W 9–10 on Depression	−0.02	0.22	−0.76
DWC W 11–12 on DWC W 9–10	0.03	0.04	0.70
DWC W 11–12 on Loneliness W 9–10	0.00	0.03	0.07
DWC W 13–14 on DWC W 11–12	0.09**	0.03	2.70
DWC W 13–14 on Loneliness W 11–12	−0.04	0.03	−1.16
RI-CLPM Loneliness-Serial 7s Model			
Loneliness W 9–10 on Age	−0.10***	0.02	−4.98
Loneliness W 9–10 on Gender	−0.06**	0.02	−3.00
Loneliness W 9–10 on Education	−0.03	0.02	−1.28
Loneliness W 9–10 on Marital status	−0.08***	0.02	−3.97
Loneliness W 9–10 on Self-health report	−0.06**	0.02	−2.83
Loneliness W 9–10 on Depression	0.10***	0.02	4.23
Loneliness W 11–12 on Loneliness W 9–10	0.11**	0.04	2.97
Loneliness W 11–12 on Se7s W 9–10	0.07	0.04	1.85
Loneliness W 13–14 on Loneliness W 11–12	0.20***	0.31	6.31
Loneliness W 13–14 on Se7s W 11–12	−0.03	0.03	−1.06
Se7s W 9–10 on Age	0.04	0.02	1.53
Se7s W 9–10 on Gender	0.01	0.02	0.55
Se7s W 9–10 on Education	−0.01	0.02	−0.69
Se7s W 9–10 on Marital status	−0.02	0.02	−1.12
Se7s W 9–10 on Self-health report	0.01	0.02	0.49
Se7s W 9–10 on Depression	0.00	0.02	0.04
Se7s W 11–12 on Se7s W 9–10	0.00	0.04	0.09
Se7s W 11–12 on Loneliness W 9–10	0.02	0.03	0.70
Se7s W 13–14 on Se7s W 11–12	0.07*	0.03	2.04
Se7s W 13–14 on Loneliness W 11–12	0.09*	0.04	2.44

***Significant at the 0.001 level. **Significant at the 0.01 level. *Significant at the 0.05 level. Loneliness W9-10, Loneliness at waves 9 and 10; Loneliness W11-12, Loneliness at waves 11 and 12; Loneliness W13-14, Loneliness at waves 13 and 14; IWC W 9–10, Immediate word recall at waves 9 and 10; IWC W 11–12, Immediate word recall at waves 11 and 12; IWC W 13–14, Immediate word recall at waves 13 and 14; DWC W 9–10, Delayed word recall at waves 9 and 10; DWC W 11–12, Delayed word recall at waves 11 and 12; DWC W 13–14, Delayed word recall at waves 13 and 14; Se7s W 9–10, Serial 7s at waves 9 and 10; Se7s W 11–12, Serial 7s at waves 11 and 12; Se7s W 13–14, Serial 7s at waves 13 and 14.

### Conditional and unconditional RI-CLPM

Six RI-CLPMs (three conditional and three unconditional with no exogenous predictors models) were computed using M*plus*. All model results were satisfactory. The loneliness and serial 7s model with age, gender, education, marital status, self-health report, and depression as predictors was the best fitting model with *χ*^2^ = 13.949, df = 13, *p* = 0.202. The unconditional model results showed that immediate word recall and loneliness also fit very well, with *χ*^2^ = 0.283, df = 1, *p* = 0.595. The RMSEA and CFI for all models were less than 0.05 and more than 0.95, respectively. TLI values always exceeded 0.95 and SRMR values were always less than 0.04 (Table 4).

**Table 4:** Fit indices in RI-CLPM s of loneliness.

Model	*Χ^2^*	*df*	*p*	CFI	TLI	SRMR	RMSEA
Loneliness-Immediate word recall (IWC) Unconditional Model RI-CLPM	0.283	1	0.595	1	1.001	0.002	0.000 [0.000, 0.019]
Loneliness-Delayed word recall (DWC) Unconditional Model RI-CLPM	0.569	1	0.451	1	1.001	0.003	0.000 [0.000, 0.021]
Loneliness-Serial 7s Unconditional Model RI-CLPM	1.962	1	0.161	1	0.998	0.005	0.009 [0.000, −0.027]
Loneliness-Immediate word recall (IWC) Model RI-CLPM	23.912	13	0.032	0.999	0.996	0.008	0.009 [0.002, 0.013]
Loneliness-Delayed word recall (DWC) Model RI-CLPM	19.464	13	0.109	0.999	0.998	0.007	0.006 [0.000, 0.012]
Loneliness-Serial 7s Model RI-CLPM	16.949	13	0.202	1	0.999	0.006	0.005 [0.000, 0.011]

### Carry-over and spill-over effects

For carry-over effects, there was a significant positive association between loneliness in each wave and loneliness in the next wave for all six models. Still, we obtained some carry-over effects for serial 7s and delayed word recall (not immediate word recall) in the conditional models. There were also some carry-over effects for all cognitive functions in the unconditional models. We found some significant spill-over effects for loneliness and cognitive function (for instance, serial 7s at waves 13–14 and loneliness at waves 11–12 and loneliness at waves 11–12 and serial 7s at waves 9–10 in the conditional model, and serial 7s at waves 13–14 and loneliness at waves 11–12 in the serial 7s unconditional model) (Tables 3, 5).

**Table 5:** Unconditional RI-CLPM Loneliness-Immediate Word Recall (IWC), Delayed Word Recall (DEC), and Serial 7s Model Results.

Standardized and unstandardized model results	*B*	SE	*β*
Unconditional RI-CLPM Loneliness-Immediate word recall (IWC) Model			
Loneliness W 11–12 on Loneliness W 9–10	0.12***	0.04	3.30
Loneliness W 11–12 on IWC W 9–10	−0.04	0.04	−1.02
Loneliness W 13–14 on Loneliness W 11–12	0.18***	0.03	5.24
Loneliness W 13–14 on IWC W 11–12	0.02	0.03	0.68
IWC W 11–12 on IWC 9–10	0.02	0.04	0.41
IWC W 11–12 on Loneliness W 9–10	−0.01	0.04	−0.23
IWC W 13–14 on IWC W 11–12	0.07**	0.03	2.03
IWC W 13–14 on Loneliness W 11–12	−0.03	0.03	−0.89
Unconditional RI-CLPM Loneliness-Delayed word recall (DWC) Model			
Loneliness W 11–12 on Loneliness W 9–10	0.12***	0.04	3.24
Loneliness W 11–12 on DWC W 9–10	−0.03	0.04	−0.92
Loneliness W 13–14 on Loneliness W 11–12	0.18***	0.03	5.20
Loneliness W 13–14 on DWC W 11–12	0.02	0.03	0.51
DWC W 11–12 on DWC W 9–10	0.03	0.04	0.67
DWC W 11–12 on Loneliness W 9–10	−0.00	0.04	−0.68
DWC W 13–14 on DWC W 11–12	0.12***	0.03	3.48
DWC W 13–14 on Loneliness W 11–12	−0.06	0.03	−1.69
Unconditional RI-CLPM Loneliness-Serial 7s Model			
Loneliness W 11–12 on Loneliness W 9–10	0.13***	0.09	3.49
Loneliness W 11–12 on Se7s W 9–10	0.06**	0.04	2.16
Loneliness W 13–14 on Loneliness W 11–12	0.18***	0.04	5.26
Loneliness W 13–14 on Se7s W 11–12	−0.03	0.03	−0.88
Se7s W 11–12 on Se7s W 9–10	−0.03	0.05	−0.51
Se7s W 11–12 on Loneliness W 9–10	0.01	0.04	0.36
Se7s W 13–14 on Se7s W 11–12	0.10**	0.04	2.54
Se7s W 13–14 on Loneliness W 11–12	0.09**	0.04	2.40

Within-person, although initially non-significant, a negative association between loneliness and immediate and delayed word recall emerged in later waves (11–12 and 13–14), with no lagged associations between heightened loneliness and diminished cognitive function observed. The conditional models indicated that older, unmarried men with low self-reported health and high depression levels were positively associated with loneliness. However, only age and self-reported health were positively linked to cognitive functions. No significant association was obtained between loneliness and cognition with education (Figures 1–6).

## Discussion

Loneliness is defined as the distress arising from deficiencies in social relationships, viewed either unidimensionally by intensity or comparatively based on past experiences or social norms (1). It impacts cognitive function directly through indicators of declining health (2) and indirectly by impairing social interactions and activities (3). Conversely, cognitive decline can predict loneliness, with research showing a significant association between loneliness and increased risk of dementia (5).

In this study, we assessed the associations between loneliness and cognitive functions in a sample of 8,473 people aged 65 or older who were evaluated repeatedly over wave 9 (2008) to wave 14 (2018) of the Health and Retirement Study (HRS). When assessing the within-person effects, we found a negative association between loneliness and immediate and delayed word recall in the later waves (11–12 and 13–14). These findings are partially consistent with the results of Cachón-Alonso et al. (11) who showed that higher levels of loneliness predicted lower cognitive function across different cognitive domains, as well as the findings of O’Luanaigh et al. (9) and Hayslip et al. (10). However, our results on first two waves (9–10 and 11–12) are consistent with those of Martin et al. (6) and Wang et al. (8) who found no substantive evidence supporting an association between loneliness and cognition, and are in line with the results of Margrett et al. (14) and Montoliu et al.’s (13) study. Our findings from the loneliness and serial 7s models in the last wave underscore the significant positive association between loneliness and serial 7s, highlighting how as individuals age, loneliness can increasingly impact their working memory. This suggests that the impact of loneliness on long and short-term memory (delayed and immediate word recall) may intensify over time, but working memory (serial 7s) may increase by increasing loneliness over time, emphasizing the need for targeted interventions in older populations. Ayalon et al. (7) also obtained the same results using the 2004, 2008, and 2012 waves in Health and Retirement Study. However, we did not find any significant lagged associations between increased loneliness and decreased cognitive function. Loneliness showed a consistent within-person effect across waves, indicating its persistence over time, while cognitive functions did not show similar persistence. On the other hand, associations from loneliness to subsequent immediate and delayed word recall were nonsignificant. However, significant associations between loneliness and later performance on serial 7s tasks were found. We consistently observed a carry-over effect for loneliness across waves for all models, but not for cognitive functions. These findings are consistent with the results reported by Cachón-Alonso et al. (11) and Zhong et al. (12).

The spill-over effects from loneliness to later immediate and delayed word recall were not significant, however, we obtained significant associations between loneliness and later serial 7s. Similarly, there was no significant association between cognitive functions and later loneliness. These findings partially confirm previous prospective studies that reported associations between loneliness and different cognitive outcomes (7, 12).

Our research, however, utilized RI-CLPMs to investigate the impact of loneliness on cognitive functions. We expanded upon Ayalon et al.’s (7) methodology by incorporating additional items from the UCLA Loneliness Scale to capture a broader understanding of loneliness. This comprehensive approach allowed us to integrate various aspects of loneliness, such as frequency of feeling alone and social attunement.

Ayalon et al.’s (7) model only partially confirmed our results. They found a negative association between memory functioning in 2004 and loneliness in 2008, as well as memory function in 2008 and loneliness in 2012, based on data from the Health and Retirement Study. However, they did not find a significant association between loneliness in the previous wave and memory functioning in the next wave. The differences in findings in these two studies could be attributed to variations in study design, sample characteristics, data collection waves, and methodological limitations. Although both studies underscore the negative correlation between loneliness and cognitive function, disparities in methodology and sample characteristics may explain the variations in specific associations observed.

We conducted three conditional RI-CLPMs to investigate the relationship between age, gender, education, marital status, self-health report, depression, and loneliness. Our findings showed a significant positive association between age and loneliness at the initial wave, which is different from the results of Dahlberg et al. (8), but consistent with the studies of Heylen (16) and Chow et al. (19). We also found significant relationships between gender and loneliness, which are consistent with the findings of Heylen (16) and Pinquart and Sorensen (17). Furthermore, we did not find any association between education and loneliness and cognitive function, which is in line with the results reported by Chow et al. (19) and Bishop and Martin (18).

Our research on the relationship between cognitive functions and covariates is consistent with previous studies conducted by Lezak (20), the Atherosclerosis Risk in Communities Study (21), and Craik and McDowd (22), all of which have shown that cognitive function tends to decline with age. However, we did not find any significant differences in cognitive functions between genders, which is contrary to the findings of the Atherosclerosis Risk in Communities Study (21), which reported that men had better cognitive function than women.

### Limitations and implications

This study had some limitations. For instance, loneliness can be influenced by numerous factors such as household numbers, work status, and economic level. These factors were not controlled in this study. Similarly, environmental and psychosocial risk factors can accelerate normal cognitive aging (30, 31), which was not accounted for in this study. Social contacts may be more significant in maintaining cognitive abilities than demographic factors such as education or gender. However, further research is needed to explore this area in more detail.

It is worth noting that previous research has shown a strong connection between loneliness and depression symptoms (32); these represent potential indirect paths through which loneliness may affect cognitive functioning (33, 34). In addition to these pathways, it is important to consider additional variables not covered in previous research to identify more factors that may influence loneliness in later life. Participants with data from six waves of the Health and Retirement Study were included in this study sample. As with any longitudinal cohort study, loss to follow-up is inevitable.

As previously discussed, Cachón-Alonso et al. (11) conducted an age-stratified analysis among 50–64 versus those older than 65 to assess the potential age differences in the associations between loneliness and cognitive function measures. In our study, we focused on individuals aged 65 and older to specifically examine the associations in a population at higher risk for cognitive decline and loneliness, which are more pronounced in older adults. However, future research should consider including a midlife age group (50–64 years) to examine age differences more comprehensively.

When examining longitudinal data within a cross-lagged framework, it is vital to distinguish between within-person and between-person levels (23). However, using the RI-CLPM also poses challenges (27). In studies examining loneliness and cognitive function over time, the RI-CLPM assumes that individuals exhibit stable trait-like loneliness levels and cognitive abilities. However, loneliness and cognitive function can fluctuate due to various factors such as life events, health changes, or social interactions. If these fluctuations are not adequately captured, the model may overestimate the stability of traits or underestimate the impact of dynamic changes in loneliness on cognitive function. In addition, loneliness and cognitive function are complex constructs influenced by both within-person changes and between-person differences. The RI-CLPM separates these levels of analysis, but interpreting within-person effects (e.g., how changes in loneliness affect cognitive function within the same individual over time) versus between-person effects (e.g., how average levels of loneliness across individuals relate to cognitive function) requires careful consideration of contextual factors and individual differences.

Using a RI-CLPM has the limitations of assuming absent inherent measurement error in single-indicator models. Measurement error can obscure true relationships between loneliness and cognitive function, leading to biased estimates of lagged effects or relationships between variables. Future studies may want to include latent variables in RI-CLPM models. Also, the assumption of stable trait variance in RI-CLPM may not apply universally across all demographic groups or contexts. For instance, older adults experiencing health declines or changes in social networks may exhibit greater variability in loneliness and cognitive function over time. Applying RI-CLPM findings from one demographic group to another without considering these differences may limit the generalizability of study results.

RI-CLPM estimates lagged effects to explore how changes in loneliness predict subsequent changes in cognitive function and vice versa. Although these estimates provide insights into temporal relationships, they do not establish causal pathways definitively. Factors beyond loneliness, such as health status, social engagement, or personality traits, could confound these relationships, necessitating cautious interpretation of lagged effects as indicative rather than causal. We need to consider these issues as the limitations of RI-CLPM, emphasizing the importance of addressing uncertainties for robust research conclusions.

Still, despite its limitations, the RI-CLPM employed in this study provided valuable insights into the relationship between loneliness and cognitive decline in a longitudinal setting. It facilitated examination of both between-person differences and within-person changes over time, enhancing understanding of developmental trajectories. Utilizing data from the Health and Retirement Study allowed replication of previous findings and robust exploration of these associations, with implications for informing policy decisions. Future research should continue to explore these relationships across diverse demographics and consider alternative modeling approaches to address the complexities inherent in longitudinal studies of loneliness and cognitive function.

## Conclusion

This study explored the relationship between loneliness and cognitive functions among over 8,000 individuals aged 65 and older, tracked over waves 9 (2008) to wave 14 (2018) of the Health and Retirement Study (HRS). We found consistent carry-over effects of loneliness across waves and some significant spill-over effects between loneliness and cognitive function. Our findings partially confirmed Cachón-Alonso et al. (11) findings and contradicted others like Martin et al.’s (6) study on Georgia’s sample, Wang et al. (8), Margrett et al. (14), and Montoliu et al.’s (13) study. All these studies did not find significant evidence supporting a link between loneliness and cognition.

## Data availability statement

Data used in this paper can be found in the Health and Retirement Study, which is located here: https://hrsdata.isr.umich.edu/data-products/rand?_gl=1*126f0s1*_ga*MTA1OTI3ODA0My4xNjY4OTkwMDc3*_ga_FF28MW3MW2*MTcyMjE5NjMwNC4zNS4xLjE3MjIxOTYzMDkuMC4wLjA.

## Ethics statement

Ethical review and approval was not required for the study of human participants in accordance with the local legislation and institutional requirements.

## Author contributions

EA: Data curation, Formal analysis, Methodology, Software, Writing – original draft. PM: Conceptualization, Formal analysis, Methodology, Software, Supervision, Writing – review & editing.
