# Supplementary material for: Unraveling the dynamics of loneliness and cognition in late life: a cross-lagged panel model
Source: Front Public Health. 2024 Aug 7;12:1425403. doi: 10.3389/fpubh.2024.1425403 (PMC11335492; doi:10.3389/fpubh.2024.1425403)
Supplement: Supplementary file 1 [file Table_1.DOCX]

Supplementary Material

Unraveling the Dynamics of Loneliness and Cognition in Late Life:

A Cross-Lagged Panel Model

**Elnaz Abaei^1*^, Peter Martin^2^**

*** Correspondence:** Corresponding Author: [abaei@iastate.edu](mailto:abaei@iastate.edu)

# Supplementary Material

*Loneliness*

Respondents were asked to indicate how much of the time felt to:

| *No.* | *Loneliness Items* | *often* | *hardly ever* | *never* |
| --- | --- | --- | --- | --- |
| 1 | lack companionship? |  |  |  |
| 2 | Left out? |  |  |  |
| 3 | Isolated from others? |  |  |  |
| 4 | That you are "in tune" with the people around you? |  |  |  |
| 5 | Alone? |  |  |  |
| 6 | That there are people you can talk to? |  |  |  |
| 7 | That there are people you can turn to? |  |  |  |
| 8 | That there are people who really understand you? |  |  |  |
| 9 | That there are people you feel close to? |  |  |  |
| 10 | Part of a group of friends? |  |  |  |
| 11 | That you have a lot in common with the people around you? |  |  |  |
